# Allergic Sensitization, Rhinitis and Tobacco Smoke Exposure in US Adults

**DOI:** 10.1371/journal.pone.0131957

**Published:** 2015-07-14

**Authors:** Josef Shargorodsky, Esther Garcia-Esquinas, Iñaki Galán, Ana Navas-Acien, Sandra Y. Lin

**Affiliations:** 1 Department of Otolaryngology-Head and Neck Surgery, Johns Hopkins University School of Medicine, Baltimore, Maryland, United States of America; 2 Coastal Ear Nose and Throat, Neptune, New Jersey, United States of America; 3 Department of Preventive Medicine and Public Health, School of Medicine, Universidad Autónoma de Madrid, Madrid, Spain; 4 National Center for Epidemiology, Carlos III Health Institute, Madrid, Spain; 5 Ciber of Epidemiology and Public Health (CIBERESP), Madrid, Spain; 6 Department of Environmental Health Sciences, Johns Hopkins University Bloomberg School of Public Health, Baltimore, Maryland, United States of America; 7 Welch Center for Prevention, Epidemiology, and Clinical Research, Johns Hopkins Medical Institutions, Baltimore, Maryland, United States of America; The Ohio State University, UNITED STATES

## Abstract

**Introduction:**

Tobacco exposure has been linked with sinonasal pathology and may be associated with allergic sensitization. This study evaluates the association between exposure to active smoking or secondhand smoke (SHS) and the prevalence of rhinitis and allergic sensitization in the US adult population.

**Methods:**

Cross-sectional study in 4,339 adults aged 20–85 in the National Health and Nutrition Examination Survey, 2005–2006. Never smoking was defined as reported lifetime smoking less than 100 cigarettes and serum cotinine levels <10ng/ml, while active smoking was defined as self-reported smoking or serum cotinine concentrations > 10 ng/mL. Self-reported rhinitis was based on symptoms during the past 12 months, and allergen sensitization was defined as a positive response to any of the 19 specific IgE antigens tested.

**Results:**

Almost half of the population (43%) had detectable levels of IgE specific to at least one inhaled allergen and 32% reported a history of rhinitis. After multivariate adjustment, there was a statistically significant association between the highest serum cotinine tertile and rhinitis in active smokers (OR 1.42; 95%CI 1.00–2.00). The association between active smoking and rhinitis was stronger in individuals without allergic sensitization (OR 2.47; 95%CI 1.44–4.23). There was a statistically significant association between increasing cotinine tertiles and decreased odds of inhaled allergen sensitization (p-trend <.01).

**Conclusion:**

Tobacco smoke exposure was associated with increased prevalence of rhinitis symptoms, but not with allergic sensitization. The results indicate that the relationship between tobacco smoke exposure and sinonasal pathology in adults may be independent of allergic sensitization.

## Introduction

Allergic sensitization is common in the US population and is a predisposing factor for multiple respiratory conditions[1]. Those conditions, including asthma and allergic rhinitis, can greatly affect an individual’s quality of life, and incur a substantial economic burden. In the United States, the total direct annual cost of allergic rhinitis alone has been shown to be as high as 3.4 billion dollars[2]. Allergic sensitization and rhinitis are very common, with a reported prevalence of rhinitis symptoms as high as 44% in the US population[3]. Despite the scope of the problem, few environmental risk factors have been identified for allergic sensitization or rhinitis.

Notwithstanding the public health efforts, tobacco smoke exposure remains common and has been linked with an increased risk of multiple upper respiratory conditions in various age groups. Significant associations have been suggested between tobacco smoke exposure and chronic sinusitis[4], asthma[5] and allergic rhinitis[6]. The data on associations between tobacco smoke exposure and allergic sensitization, however, have been conflicting. Some studies have reported significant associations between IgE mediated allergic sensitization and tobacco smoke[7], while others have reported either no association[8] or even an inverse association between allergic sensitization and tobacco smoke exposure[9]. This suggests that tobacco exposure may predispose individuals to rhinitis, but the mechanism may or may not involve an increased predisposition to allergic sensitization.

Despite the high incidence of both, tobacco exposure and multiple upper respiratory ailments, there is still no clear understanding of the relation between tobacco exposure, allergic sensitization, and rhinitis. Large-scale epidemiologic studies evaluating the associations between smoking exposure and these two conditions are lacking in the literature. Therefore, this study aims to evaluate the association between exposure to active smoking or secondhand smoke (SHS) and the prevalence of rhinitis and allergic sensitization in the US adult population.

## Materials and Methods

Between 2005–2006, the National Health and Nutrition Examination Survey (NHANES) examined a US nationally representative sample by using a complex multistage sample design. NHANES provides nationally representative cross-sectional data of the health status of the civilian, non-institutionalized US population. After selection using a complex survey design, participants were interviewed and examined. Older individuals, Mexican Americans and black individuals were intentionally oversampled. Therefore, appropriate sample weights were used to obtain weighted regression estimates, and the final results of our analyses are generalizable to the US population[10]. The NHANES protocol was reviewed and approved by the National Center for Health Statistics Institutional Review Board. Written informed consent was given by the participants for their clinical records to be used in a research study. The unweighted response rate for adult participants during the NHANES 2005–2006 was 74%[11].

### Study Population

For this study, we selected individuals ≥20 years of age who participated in the NHANES 2005–2006 (N = 4,979). We then excluded participants with missing values in serum cotinine (N = 495), allergic sensitization (N = 70) or rhinitis (N = 1), as well as those without information on education level (N = 5) or BMI (N = 69). Participants included in the analyses were similar with respect to the main socio-demographic variables when compared with the original sample of adults surveyed (data not shown).

### Exposure Assessment

Serum cotinine is a metabolic byproduct of nicotine, and has been demonstrated as an accurate measure of recent nicotine exposure. The half-life of cotinine is approximately 17 hours, allowing time for detection of recent nicotine exposure[12]. Serum cotinine was measured by an isotope dilution-high performance liquid chromatography/atmospheric pressure chemical ionization tandem mass spectrometry [CDC]. The limit of detection (LOD) for serum cotinine was 0.015 ng/mL. Serum cotinine concentrations below the LOD were replaced by the LOD divided by the square root of 2. Because serum cotinine only allows for measurement of recent nicotine exposure, past exposure data must still be obtained from self-report. Tobacco use and exposure to second-hand smoke were assessed by using self-reported information and serum cotinine. Participants were analyzed in two categories: Secondhand smoke (SHS) exposure and active smoking. Within the SHS analysis, individuals who reported never smoking and had serum cotinine levels below the level of detection were categorized as unexposed. Those who reported never smoking and had serum cotinine levels above the level of detection, but < = 10ng/ml were divided into tertiles of serum cotinine. Within the active smoking analyses, individuals who reported smoking less than 100 cigarettes in their life and had serum cotinine levels <10ng/ml were classified as never smokers. Those who reported smoking more than 100 cigarettes in their life but none in the last month and had serum cotinine <10ng/ml were classified as former smokers. The participants with serum cotinine > = 10ng/ml were then divided into tertiles of serum cotinine levels.

### Outcome Assessment

NHANES 2005–2006 collected information on allergic symptoms and sensitization. Serum samples were analyzed for 19 allergen specific IgEs *(Dermatophagoides farinae*, *Dermatophagoides pteronyssinus*, cat, dog, cockroach, *Alternaria alternata*, peanut, egg, cow’s milk, ragweed, rye grass, bermuda grass, oak, birch, shrimp, *Aspergillus fumigatus*, Russian thistle, mouse and rat) using the Pharmacia Diagnostics ImmunoCAP 1000 System (Kalamazoo, MI, USA). The lower limit of detection for each of the allergen-specific IgE antibody tests was 0.35 kU/L. Allergen sensitization was defined as a positive response if the level of any of the 19 specific IgE antigens was above the lower limit of detection (0.35 kU/L). Additionally inhaled allergen sensitization was defined as a positive response to Dermatophagoides farina, Dermatophagoides pteronyssinus, cat, dog, cockroach, alternaria alternata, ragweed, rye grass, Bermuda grass, oak, birch, Aspergillus fumigatus, Russian thistle, mouse, rat and food allergen sensitization as a positive response to peanut, egg, cow's milk or shrimp specific IgEs. Self-reported rhinitis was defined as an affirmative reply to the question “during the last 12 months have you had a problem with sneezing, or a runny, or blocked nose when you did not have a cold or the flu?”

### Adjustment variables

Questionnaire information included gender, age, education (less than high school, high school, and more than high school), race/ethnicity (non-Hispanic white, non-Hispanic black, Mexican American, other) and BMI (<25, 25–30, >30).

### Statistics

Statistical analyses were performed in STATA version 11.2 statistical software (Stata Corp, College Station, TX) by using the survey (svy) command to account for the complex sampling design and weights in the NHANES.

The prevalence of allergic sensitization, inhalant allergen sensitization, food allergen sensitization and rhinitis was calculated overall and by sex, age, ethnicity, parental education and BMI percentiles. We also estimated odds ratios (OR) and their 95% confidence intervals (95%CI) to assess the association between tobacco smoke exposure and the studied outcome variables using logistic regression models. The relation between tobacco smoke exposure and self-reported rhinitis, independent of allergic sensitization, was further explored by conducting a separate analysis of only the individuals with no allergic sensitization. P-trend values were computed by assigning the median concentration in each tertile to participants and evaluating this as a continuous variable.

## Results

A total of 4,339 individuals were included in the study. As shown in Table 1, approximately 43% of participants (N = 1,865) were sensitized to at least one aeroallergen and 32% (N = 1,409) self-reported having rhinitis. Individuals who were male, black and in the youngest age group were more likely to have allergic sensitization. *Individuals who were male*, *black and in the youngest age group were more likely to have allergic sensitization*. *Individuals with the lowest education level were positive for fewer inhaled allergens*, *but more food allergens*. *Female*, *white individuals*, *and individuals with greater than high school education showed a higher prevalence of rhinitis*. There were no apparent differences in rhinitis or allergic sensitization prevalence between the education or BMI categories.

**Table 1:** Allergic sensitization and self-reported rhinitis by participant characteristics in US adults (N = 4,339).

		AS—Inhaled		AS—Food		AS–Inhaled and Food		Self-reported Rhinitis	
		+	-	+	-	+	-	+	-
		(N = 1865)	(N = 2474)	(N = 721)	(N = 3618)	(N = 637)	(N = 3702)	(N = 1409)	(N = 2930)
Gender	Male	54.8	43.1	60.2	45.9	62.8	45.8	44.9	49.8
	Female	45.2	56.9	40.8	54.1	37.2	54.2	55.1	50.2
Age	20–38	45.5	30.4	38.7	34.9	39.5	34.9	33.4	36.6
	39–53	32.4	31.2	31.1	31.8	32.8	31.5	34.2	30.3
	54–85	25.1	38.4	30.2	33.3	27.7	33.6	32.4	33.1
Ethnicity	White	67.9	75.7	61	74.4	59.8	74.3	78.9	68.8
	Black	14.1	8.8	18.1	9.8	19.3	9.8	8.6	12.4
	Mexican	8.4	7.9	9.8	7.8	10.2	7.8	5.3	9.6
	Other	9.6	7.6	1.1	8	10.7	8.1	7.2	9.2
Education	<HS	15.3	19.1	20	17	19.2	17.2	13	20
	HS	24.2	25.2	24.3	24.9	24.2	24.9	22.4	26.1
	>HS	60.5	55.7	55.7	58.1	56.6	57.9	64.6	53.9
BMI	Normal	33.1	32.6	29.3	33.5	30.7	33.2	31.7	33.4
	Overweight	33.7	32.5	34.5	32.7	34.1	32.8	32.3	33.3
	Obese	33.2	34.9	36.1	33.8	35.2	34	36	33.2

Results for the association between allergic sensitization, self-reported rhinitis and SHS exposure are shown in Table 2. Three models are displayed: a univariate model (model 1), a multivariate model adjusted for age and gender (model 2) and a multivariate model adjusted for age, gender, ethnicity, education and BMI (model 3). A significant trend was apparent between increasing cotinine levels and increased unadjusted odds of inhaled and food allergic sensitization. However, this relation did not remain significant after multivariate adjustment. There were no significant associations between SHS exposure categories and rhinitis. In a similar fashion, Table 3 shows the results for the active smoking analyses. Increasing exposure to active smoking was significantly associated with decreased odds of inhaled allergen sensitization in all 3 models (p-trend = .03 in model 1 and p-trend < .01 in models 2 and 3). Conversely, the highest tertile of serum cotinine was significantly associated with increased multivariable-adjusted odds of self-reported rhinitis (OR 1.42; 95%CI 1.00–2.00). The trend, however, was not significant in this analysis.

**Table 2:** Odds ratios and 95% confidence intervals for the association between tertiles of secondhand smoke (SHS) exposure, allergic sensitization and rhinitis.

			ALLERGIC SENSITIZ INHALED			ALLERGIC SENSITIZ FOOD			ALLERGIC SENSITIZ INHALED+FOOD			RHINITIS SELF-REPORT		
		N	Model 1	Model 2	Model 3	Model 1	Model 2	Model 3	Model 1	Model 2	Model 3	Model 1	Model 2	Model 3
**Unexposed**														
	<0.011	778	1	1	1	1	1	1	1	1	1	1	1	1
**Serum cotinine (ng/ml)**														
	0.011–0.031		0.96	0.93	0.86	1.22	1.4	1.08	1.21	1.12	1.06	0.87	0.89	0.92
	0.011–0.031	804	(0.85–1.12)	(0.81–1.08)	(0.66–1.12)	(0.88–1.69)	(0.85–1.55)	(0.81–1.46)	(0.81–1.81)	(0.77–1.62)	(0.74–1.53)	(0.65–1.16)	(0.66–1.19)	(0.67–1.27)
	0.032–0.086		1.07	1	0.99	1.36	1.24	1.15	1.43	1.27	1.19	0.99	1.02	1.09
	0.032–0.086	724	(0.85–1.12)	(0.86–1.17)	(0.71–1.40)	(0.97–1.89)	(0.91–1.69)	(0.82–1.60)	(0.94–2.17)	(0.84–1.92)	(0.76–1.85)	(0.74–1.33)	(0.76–1.36)	(0.79–1.49)
	0.087–10		1.07	0.97	0.91	1.5	1.29	1.16	**1.66**	1.37	1.24	0.82	0.86	0.97
	0.087–10	848	(0.89–1.29)	(0.80–1.17)	(0.61–1.36)	(0.99–2.26)	(0.87–1.91)	(0.77–1.74)	**(1.05–2.62)**	(0.87–2.16)	(0.78–1.98)	(0.64–1.06)	(0.67–1.10)	(0.75–1.26)
p-trend			0.95	0.99	0.84	0.07	0.25	0.51	0.03	0.17	0.35	0.21	0.37	0.8

**Table 3:** Odds ratios and 95% confidence intervals for the association between active tobacco smoking, allergic sensitization and rhinitis.

			ALLERGIC SENSITIZ INHALED				ALLERGIC SENSITIZ FOOD			ALLERGIC SENSITIZ INHALED+FOOD			RHINITIS SELF-REPORT		
		N	Model 1		Model 2	Model 3	Model 1	Model 2	Model 3	Model 1	Model 2	Model 3	Model 1	Model 2	Model 3
**Never**		2172		1	1	1	1	1	1	1	1	1	1	1	1
**Former**		982	**0.76**		0.83	0.87	0.91	0.9	0.97	0.92	0.94	1.02	1.08	1.09	1.02
			**(0.60–0.95)**		(0.64–1.06)	(0.67–1.10)	(0.72–1.16)	(0.70–1.16)	(0.76–1.26)	(0.72–1.21)	(0.70–1.26)	(0.76–1.38)	(0.87–1.33)	(0.788–1.36)	(0.82–1.27)
**Current**															
	10–143	442	0.97		0.77	0.8	1.1	0.94	0.97	1.17	0.96	0.99	0.76	0.81	0.85
			(0.71–1.31)		(0.56–1.06)	(0.58–1.11)	(0.80–1.53)	(0.69–1.30)	(0.71–1.33)	(0.79–1.74)	(0.66–1.41)	(0.68–1.44)	(0.87–1.33)	(0.63–1.03)	(0.67–1.08)
	144–284	377	**0.75**		**0.64**	**0.68**	0.91	0.8	0.85	1.03	0.88	0.95	0.97	1.01	1.08
			**(0.61–0.92)**		**(0.52–0.80)**	**(0.54–0.86)**	(0.59–1.39)	(0.51–1.26)	(0.52–1.36)	(0.66–1.60)	(0.55–1.42)	(0.56–1.62)	(0.68–1.38)	(0.70–1.47)	(0.74–1.57)
	≥285	366	**0.8**		**0.67**	**0.71**	1.31	1.15	1.23	1.34	1.15	1.23	1.28	1.34	**1.42**
			**(0.59–0.92)**		**(0.51–0.93)**	**(0.51–0.98)**	(0.92–1.87)	(0.81–1.62)	(0.83–1.85)	(0.93–1.92)	(0.81–1.63)	(0.81–1.90)	(0.91–1.79)	(0.96–1.43)	**(1.00–2.00)**
p-trend			**0.03**		**<0.01**	**<0.01**	0.19	0.62	0.96	0.11	0.77	0.5	0.57	0.33	0.18

Serum cotinine in ng/ml

Never smokers: participants who self-reported (“I have not smoked more than 100 cigarettes in life”) + did not have serum cotinine levels > = 10 ng/mL

Former smokers: participants who self-reported (“I have smoked more than 100 cig in life but I haven’t smoked in the last month”) + did not have serum cotinine levels > = 10 ng/mL

Current smokers: participants who self-reported (“I have smoked more than 100 cig in life and I have smoked in the last month”) OR had serum cotinine levels > = 10 ng/mL

In order to further evaluate the relation between tobacco smoke exposure and rhinitis, independent of allergic sensitization, we performed stratified analyses. Tables 4 and 5 show the analyses of the relations between tobacco smoke exposure and rhinitis stratified by allergic sensitization. Analyses of rhinitis and SHS are shown in Table 4, and those of rhinitis and active smoking are shown in Table 5. A significant trend was observed between increasing cotinine levels and decreasing odds of rhinitis in allergic sensitized individuals (p = .05). The interaction of SHS by sensitization categories, however, was not statistically significant (p-interaction = .24). Non-sensitized individuals showed a significant trend of increasing multivariate-adjusted odds of rhinitis with increasing serum cotinine levels (p < .01). Non-sensitized individuals in the highest cotinine tertile had significantly higher odds of rhinitis than never-smokers (OR 2.47; 95%CI 1.44–4.23). The p-interaction between the allergic sensitization status and active smoking the relation between tobacco smoke exposure and rhinitis was statistically significant (p < .01).

**Table 4:** Odds ratio and 95% confidence intervals for the association between secondhand smoke exposure and rhinitis by sensitization status.

		Non sensitized adults				Sensitized adults				
		N	Model 1	Model 2	Model 3	N	Model 1	Model 2	Model 3	p-Interaction*
**Unexposed**		432	1	1	1	346	1	1	1	
**SHS**										
	0.011–0.031	461	1.2	1.25	1.4	343	**0.57**	**0.57**	0.91	
			(0.86;1.91)	(0.92;2.00)	(0.92;2.12)		**(0.35;0.91)**	**(0.36;0.92)**	(0.37;1.01)	
	0.032–0.086	382	1.29	1.4	1.48	342	0.72	0.73	0.8	
			(0.85;1.97)	(0.92;2.13)	(0.94;2.32)		(0.49;1.04)	(0.50;1.07)	(0.56;1.16)	
	0.087–10	446	1.02	1.15	1.31	402	**0.62**	0.63	0.73	
			(0.68;1.55)	(0.75;1.75)	(0.82;2.10)		**(0.40–0.94)**	(0.41;0.97)	(0.47;1.14)	
p-trend			0.91	0.47	0.2		**0.05**	0.08	0.28	0.24

*P interaction base on model 3

**Table 5:** Odds ratio and 95% confidence intervals for the association between active tobacco smoking and rhinitis by sensitization status.

		Non sensitized adults				Sensitized adults				
		N	Model 1	Model 2	Model 3	N	Model 1	Model 2	Model 3	p-interaction*
**Never**		1136	1	1	1	1036	1	1	1	
**Ever**		585	1.21	1.2	1.12	397	1.05	1.07	1	
			(1.00–1.47)	(0.97–1.50)	(0.91–1.38)		(0.75–1.46)	(0.77–1.47)	(0.72;1.41)	
**Current**										
	10–143	237	0.96	1.29	1.15	205	0.62	0.63	0.69	
			(0.71–1.27)	(0.88–1.88)	(0.83–1.59)		(0.40–0.95)	(0.42–0.96)	(0.46–1.02)	
	144–284	223	1.18	1.29	1.35	154	0.85	0.88	0.96	
			(0.83–1.70)	(0.88–1.88)	(0.88–2.07)		(0.51–1.43)	(0.52–1.49)	(0.57–1.61)	
	≥285	209	**2.06**	**2.29**	**2.47**	157	0.74	0.77	0.78	
			**(1.24–3.43)**	**(1.17–1.88)**	**(1.44–4.23)**		(0.48–1.15)	(0.93–1.43)	(0.54–1.11)	
p-trend			0.02	**<0.01**	**<0.01**		0.09	0.09	0.13	**<0.01**

*P interaction base on model 3

Multiple sensitivity analyses were also performed. Results remained consistent when participants who had smoked during the 12 months prior to the study were considered as current smokers. In addition, adjusting the multivariable analyses for pack years of smoking did not change the results, nor did the adjusting for the presence of chronic obstructive pulmonary disease (COPD). A similar analysis to Table 5 performed in participants with a positive history of asthma, showed a somewhat stronger association between rhinitis and smoking in non-sensitized adults, suggesting a possibility that non-allergic individuals with both asthma and rhinitis may be prone to an even greater respiratory mucosal inflammatory response to tobacco smoke than those without asthma.

## Discussion

This study evaluated the association between exposure to active smoking or SHS and the prevalence of rhinitis and allergic sensitization in the US adult population. The prevalence of rhinitis in our cohort was consistent with previous reports utilizing NHANES data[13,14] as well as with other US nationwide surveys[3]. The assessment of tobacco smoke exposure in this study was highly sensitive, assigning tobacco exposure to individuals with even very low levels of serum cotinine. This method of assessing tobacco exposure in the NHANES dataset has been previously described and the prevalence of exposure in this study was consistent with prior reports[15]. Individuals identified as active smokers had a significantly higher prevalence of rhinitis. This relationship did not appear to be due to allergic sensitization, as tobacco smoke exposure was not significantly associated with allergic sensitization in the multivariate-adjusted models. Sensitization to inhaled allergens was actually inversely related to cotinine levels in active smokers. In addition, analyses stratified by sensitization status showed even stronger associations between active tobacco smoke exposure and rhinitis among non-sensitized participants.

Associations between tobacco smoke exposure and multiple respiratory diseases have previously been documented in the literature. In a 2011 study of 200 individuals, current as well as past SHS exposure was found to be a significant risk factor for allergic rhinitis[16]. Likewise, both active smoking and SHS have demonstrated a significant association with an increased prevalence of chronic rhinosinusitis[4]. It has long been evident that smoking exposure is associated with worse pulmonary function test results[17], but tobacco exposure has also been shown to be associated with asthma[18], bronchitis[19], and chronic cough[20]. A 2006 study of 77 bar employees prospectively evaluated before and after the introduction of a smoking ban noted a significant improvement in multiple respiratory health measures following the ban[21]. Tobacco smoke exposure has been shown to modify the physiology of the normal respiratory tract, with demonstrated thickening of the lower airway walls, impairment of mucociliary clearance, and alteration of the airway immune function[22]. The specific mechanism for the relation between rhinitis and tobacco smoke exposure, however, has not been proven.

Although there is concern that allergic sensitization is a potential mechanism for development of rhinitis in individuals exposed to tobacco smoke, our study found that the association between rhinitis prevalence and tobacco smoke exposure may be independent of allergic sensitization. This is consistent with other recent findings. A cross-sectional study of 18,087 individuals in Sweden found a higher prevalence of allergic rhinitis associated with nonsmokers than smokers, but a higher prevalence of chronic sinusitis and nasal congestion associated with a positive smoking history[23]. These findings offer evidence that the mechanism linking smoking and IgE mediated allergic sensitization may be distinct from those linking smoking with rhinitis.

The relationship between allergic sensitization and tobacco smoke exposure is complex. Past studies have observed significant associations between tobacco smoke and increased allergic sensitization. On the other hand, evidence has also been shown for no association, or even potentially reverse relationship between smoking exposure and mean IgE measurements[9]. A 32 year longitudinal study in New Zealand published in 2008 demonstrated no significant association between smoking exposure and allergic sensitization[24]. In addition, lower absolute eosinophil counts have been observed in active smokers compared to never-smokers[25]. Results from studies looking at chronic airway inflammation have also suggested distinct eosinophilic and non-eosinophilic phenotypes of inflammation, with potentially distinct clinical disease courses and responses to treatment[26]. One mechanism for the relation between rhinitis and smoking may be neurogenic inflammation induced by cigarette smoke. Neurogenic inflammation resulting from environmental exposures has been described in the literature and represents an inflammatory mechanism distinct from that of allergic sensitization[27]. The non-allergic etiology of nasal inflammation is further supported by multiple studies showing a lack of association between aeroallergen sensitization and tobacco smoke exposure[8,28]. The physiologic mechanism of the association observed in our study may also be the result of immunosuppressive effects of tobacco smoke, as past work has demonstrated inhibition of pro-inflammatory cytokines[29], reduction in serum levels of immunoglobulins, and inhibition of T-cell activation[30] in tobacco-exposed individuals. In fact, it may be this same inflammatory inhibition that leads to the documented increased incidence of various infections in individuals of all ages[31,32]. These mechanisms may explain the difference in the associations with tobacco exposure between self-reported rhinitis and allergic sensitization in our study.

The strengths and limitations of our study should be considered. Data from NHANES are comprehensive and nationally representative, drawing from a large and diverse sample of participants. The NHANES tobacco smoke exposure measures have been shown to be objective and reliable in past studies[15]. The study, however, is cross-sectional, and therefore causality with respect to exposure variables cannot be determined. Rhinitis was self-reported, and so may be prone to recall bias. However, we expect that recall bias would be similar in participants exposed and those unexposed to tobacco smoke. Although multivariate adjusted models were utilized in the analyses, there is also the possibility of residual confounding by unmeasured factors. Reverse causation may also play a role, as individuals diagnosed with allergic sensitization or respiratory conditions potentially actively avoiding further tobacco exposure. In our study population, however, the prevalence of smoking exposure was high, and with a wide distribution of cotinine measurements, making this phenomenon less likely to affect the study outcomes.

## Conclusions

This study demonstrated a significantly higher prevalence of self-reported rhinitis symptoms in adult smokers, and this was observed to be independent of allergic sensitization. The results of this study highlight the complex relationship between tobacco smoke exposure and sinonasal pathology, and suggest that the effect of smoking exposure on rhinitis symptoms may have a mechanism distinct from allergic sensitization. Regardless of the mechanism, however, the observed associations between rhinitis and smoking provide further evidence for the importance of minimizing tobacco smoke exposure.
